# Strong controls of daily minimum temperature on the autumn photosynthetic phenology of subtropical vegetation in China

**DOI:** 10.1186/s40663-021-00309-9

**Published:** 2021-05-19

**Authors:** Peixin Ren, Zelin Liu, Xiaolu Zhou, Changhui Peng, Jingfeng Xiao, Songhan Wang, Xing Li, Peng Li

**Affiliations:** 1 College of Geographic Science, Hunan Normal University, Changsha, 410081 China; 2 Department of Biology Sciences, Institute of Environment Sciences, University of Quebec at Montreal, C.P. 8888, Succ. Centre-Ville, Montreal, H3C 3P8 Canada; 3 Earth Systems Research Center, Institute for the Study of Earth, Oceans, and Space, University of New Hampshire, Durham, NH 03824 USA; 4 International Institute for Earth System Sciences, Nanjing University, Nanjing, 210023 China; 5 Jiangsu Provincial Key Laboratory of Geographic Information Technology, Key Laboratory for Land Satellite Remote Sensing Applications of Ministry of Natural Resources, School of Geography and Ocean Science, Nanjing University, Nanjing, 210023 China

**Keywords:** Carbon cycle, Evergreen vegetation, Plant phenology, Solar-induced Fluorescence, Climate change, MODIS, Eddy covariance

## Abstract

**Background:**

Vegetation phenology research has largely focused on temperate deciduous forests, thus limiting our understanding of the response of evergreen vegetation to climate change in tropical and subtropical regions.

**Results:**

Using satellite solar-induced chlorophyll fluorescence (SIF) and MODIS enhanced vegetation index (EVI) data, we applied two methods to evaluate temporal and spatial patterns of the end of the growing season (EGS) in subtropical vegetation in China, and analyze the dependence of EGS on preseason maximum and minimum temperatures as well as cumulative precipitation. Our results indicated that the averaged EGS derived from the SIF and EVI based on the two methods (dynamic threshold method and derivative method) was later than that derived from gross primary productivity (GPP) based on the eddy covariance technique, and the time-lag for EGS_sif_ and EGS_evi_ was approximately 2 weeks and 4 weeks, respectively. We found that EGS was positively correlated with preseason minimum temperature and cumulative precipitation (accounting for more than 73% and 62% of the study areas, respectively), but negatively correlated with preseason maximum temperature (accounting for more than 59% of the study areas). In addition, EGS was more sensitive to the changes in the preseason minimum temperature than to other climatic factors, and an increase in the preseason minimum temperature significantly delayed the EGS in evergreen forests, shrub and grassland.

**Conclusions:**

Our results indicated that the SIF outperformed traditional vegetation indices in capturing the autumn photosynthetic phenology of evergreen forest in the subtropical region of China. We found that minimum temperature plays a significant role in determining autumn photosynthetic phenology in the study region. These findings contribute to improving our understanding of the response of the EGS to climate change in subtropical vegetation of China, and provide a new perspective for accurately evaluating the role played by evergreen vegetation in the regional carbon budget.

**Supplementary Information:**

The online version contains supplementary material available at 10.1186/s40663-021-00309-9.

## Introduction

Vegetation phenology refers to the rhythm of growth and development in the life cycle of plants, which is closely associated with seasonal changes in the environment (Suepa et al. 2016). The life cycle comprises biological events that occur throughout the year such as budding, flowering, fruiting, defoliation and dormancy during the growth of plants in a year (Helmut 1974; Vrieling et al. 2018). Vegetation phenology is a key indicator of climate change, and it can have a significant impact on the cycles of carbon, water, and energy in terrestrial ecosystems (Xiao et al. 2009; Richardson et al. 2013). Apart from gaining a better understanding of plant responses to climate change, studies on vegetation phenology can help us better understand the mechanisms involved in the exchange of matter and energy between vegetation and the atmosphere, and more accurately evaluate the contribution of vegetation to the global carbon budget (Piao et al. 2008; Penuelas et al. 2009; Richardson et al. 2013).

A large number of studies based on ground observations and satellite remote sensing monitoring have reported an advance in the start of vegetation growing season (SOS) during spring (Menzel et al. 2006; Fu et al. 2015) and a delay in the end of growing season (EGS) in autumn (Liu et al. 2016; Piao et al. 2019). Evidence from multiple studies indicated earlier SOS was significantly related to the increase of preseason temperature (Ge et al. 2015; Xu et al. 2018). Compared with SOS, the EGS also determines the length of the entire growing season and plays an important role in maintaining the global carbon balance (Piao et al. 2008; Garonna et al. 2014). However, the mechanisms underlying the response of EGS to climate change remain unclear (Gallinat et al. 2015; Wu et al. 2018). Though multiple studies tried to investigate the relationships between EGS and climate factors, there is still no consistent conclusion (Yang et al. 2017; Wu et al. 2018). For example, recent studies revealed that increase in daytime maximum temperature and nighttime minimum temperature would cause contrasting effects on drought stress, which result in inconsistent relation between autumn vegetation phenology with preseason maximum temperature and minimum temperature (Wu et al. 2018).

Previous studies on vegetation phenology have focused on the deciduous forest in the middle and high latitudes (Fu et al. 2014; Flynn and Wolkovich 2018), while fewer studies have been conducted in subtropical regions with evergreen vegetation. Remote sensing based indicators have been frequently applied in vegetation phenology monitoring, such as Normalized Difference Vegetation Index (NDVI) and Enhanced Vegetation Index (EVI) (Wen et al. 2017; Yuan et al. 2018). However, these vegetation indices based on “greenness” observations can only reflect the greenness information of vegetation, and represent the “potential photosynthesis” of vegetation (Liu et al. 2018). Subtropical regions mainly consist of evergreen forests and do not show significant seasonal changes with respect to canopy “greenness”. Therefore, using vegetation index to understand vegetation phenology and the response mechanism of vegetation phenology to climate change in these forests can cause significant bias (Karkauskaite et al. 2017).

The emergence of solar-induced chlorophyll fluorescence (SIF) in the recent decade provides a new opportunity to monitor vegetation phenology from regional to global scales (Guanter et al. 2014; Yoshida et al. 2015; Sun et al. 2017). SIF is measured as light released during photosynthesis between the wavelengths of 650–800 nm, which can directly reflect the dynamic changes of plant photosynthesis (Frankenberg et al. 2014). Compared to traditional vegetation indices that are significantly affected by atmospheric aerosols, soil, snow, and clouds (Zhang et al. 2003; Balzarolo et al. 2016; Liu et al. 2018), SIF can more directly reflect the dynamic changes that occur during photosynthesis and is less sensitive to the influence of clouds or the atmosphere (Joiner et al. 2014). Therefore, SIF is considered to be a reliable remote sensing-based indicator for monitoring the phenology of tropical or subtropical evergreen vegetation, especially in forests that are in the early stages of environmental stress (Köhler et al. 2018; Zuromski et al. 2018). In addition, SIF was also considered more effective in retrieving vegetation phenology in high productivity areas (Guanter et al. 2014; Yang et al. 2015).

A quarter of the land area in China is covered by subtropical forests, characterized by unique vegetation types, high biodiversity, and remarkable ecological functions. These forests play an important role in maintaining the ecological balance in the region. However, there were still few studies on the phenology of subtropical vegetation, especially in EGS. Therefore, understanding the response of subtropical vegetation to climate change is critical, especially in terms of autumn phenology. In this study, we examined the autumn photosynthetic phenology for five vegetation types in subtropical China, including evergreen coniferous forest (ECF), evergreen broadleaved forest (EBF), deciduous broadleaved forest (DBF), shrub and grassland. The SIF, NIRv (near-infrared reflectance of vegetation) and MODIS EVI remote sensing data from 2000 to 2018 were used to extract the autumn phenology of subtropical vegetation in China. The main objectives of the study were as follows: (1) to examine the spatiotemporal patterns of the EGS in the study region; (2) to compare the results of vegetation photosynthetic phenology based on SIF and vegetation index; (3) to analyze the responses of vegetation autumn phenology in subtropical China to maximum temperature (*T*_max_), minimum temperature (*T*_min_) and precipitation and then to explore the underlying mechanisms.

## Materials and methods

### Datasets

#### Remote sensing data

The satellite SIF data used in this study is provided by Xiao (2019), which is a global high spatio-temporal resolution (0.05°, 8-day) SIF dataset (namely, GOSIF) based on the OCO-2 (Orbiting Carbon Observatory 2) satellite. GOSIF was developed by a data-driven method which established a predictive SIF model from discrete OCO-2 soundings, MODIS remote sensing data and meteorological reanalysis data (Li and Xiao 2019a). It has similar and reasonable seasonal period as the original OCO-2 SIF, but it has higher spatial and temporal resolution, global continuous coverage and longer data record (Li and Xiao 2019a). This dataset plays an important role in understanding the long-term trends in global photosynthesis, and which has been widely used to evaluate the inter-annual variation in ecosystem productivity (Li and Xiao 2019b; Li and Xiao 2020; Qiu et al. 2020). In calculation, we changed the temporal resolution of SIF data from 8 to 16 days by using the maximum synthesis method. The EVI data used in this study were extracted from NASA Earth Science Data (NASA 2015). For the analysis, we used EVI data (2000–2018) extracted from the MOD13C1 v006 dataset, with a spatial resolution of 0.05° and 16 days’ interval. The NIRv was calculated by the product of normalized vegetation index (NDVI) and near infrared reflectance, with a spatial resolution of 0.05 degree (Wang et al. 2021).

#### Meteorological data

Meteorological data were obtained from the National Tibetan Plateau Data Center (2019). The dataset is based on the fusion of remote sensing product, reanalysis data set and field station data and has a temporal resolution of 3 h and a spatial resolution of 0.1° (Yang et al. 2010). The dataset provides seven near-surface meteorological elements, including air temperature, surface pressure, specific humidity, wind speed, downward shortwave radiation, downward long-wave radiation and precipitation rate (He et al. 2020). In this study, we used daily precipitation, *T*_max_ and *T*_min_ data to examine the response of EGS to climate change.

#### Flux and vegetation data

**Fig. 1:**
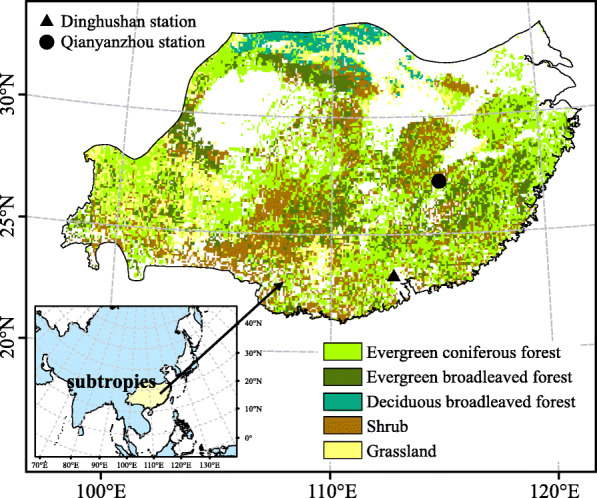
The spatial distribution of vegetation types and flux stations in study area

In this study, carbon flux data of half hour scale in terrestrial ecosystem were used to evaluate the performance of different remote sensing data for monitoring vegetation phenology. The flux data of the Dinghushan and Qianyanzhou research stations from 2003 to 2010 were obtained from the national flux network of China (ChinaFLUX 2013). Coordinate axis rotation and WPL (Webb-Pearman-Leuning) correction were used to eliminate the effects of topography, air hydrothermal transmission, and observation height on the observed data. Then, the CO_2_ flux data were partitioned into gross primary productivity (GPP) and total ecosystem respiration. The vegetation data (spatial resolution, 1 km) were obtained from the Joint Research Center of European Commission under the project of Global Land Cover 2000. The final regional vegetation classification data for China were obtained by preprocessing the corresponding data (Xu et al. 2005). We excluded cultivated areas affected by anthropogenic activity. For the final analysis, we selected five vegetation types in the subtropical region of China, including evergreen coniferous forest (ECF), evergreen broadleaved forest (EBF), deciduous broadleaved forest (DBF), shrub and grassland. The distribution of the subtropical region and vegetation type were shown in Fig. 1.

### Estimation of EGS

In order to eliminate the background noise, the Savitzky-Golay filter was applied to smooth the SIF, EVI, NIRv and GPP flux time series data (Zhang et al. 2016). We then used the dynamic threshold method and the derivative method to determine EGS, which indicates autumn vegetation phenology (Liu et al. 2016; Filippa et al. 2016). Compared with the fixed threshold method, the dynamic threshold method is advantageous since it eliminates the influence of background noise by allowing a threshold to be set based on the conditions in the study area (White et al. 2009). The equation of the dynamic threshold method is as follows:

1 $$ {X}_{\mathrm{ratio}}=\frac{X_t-{X}_{\mathrm{min}}}{X_{\mathrm{max}}-{X}_{\mathrm{min}}} $$

where *X*_*t*_ is the value of *X* at a given time *t*, and *X*_max_ and *X*_min_ are the maximum and minimum values of *X* in the annual *X* time series, respectively. *X* indicates SIF, EVI and NIRv remote sensing data. In this study, the EGS date was defined as the first day of the year in the descending period when the *X*_ratio_ reduction value was less than 0.5 as the EGS (Wu et al. 2018).

The derivative method assumes that the EGS is the time point when the *X* time series decreases rapidly: this corresponds to the points with the minimum slope of the fitting curve (Forkel et al. 2015). Here the *X*_ratio_ is calculated as the change in *X* at time *t*, based on the following equation:

2 $$ {X}_{\mathrm{ratio}}=\frac{X_{\left(t+\Delta  t\right)}-{X}_t}{\Delta  t} $$

where *X*_*t*_ represents the value of *X* corresponding to the time *t*. ∆*t* is value of the time variation. *X* indicates SIF, EVI and NIRv remote sensing data. In order to make the retrieval phenology more accurate, EGS retrieved from two methods was both used for the analysis.

### Analysis

We first extracted the autumn photosynthetic phenology (i.e., EGS) for each year at each pixel using both derivative and dynamic threshold methods from the GOSIF, NIRv and MODIS EVI datasets for the period from 2000 to 2018. We then calculated the annual average of EGS and analyzed its spatial distribution in the subtropical region. Subsequently, we used flux data to retrieve the annual EGS by the two methods in the study area, in order to evaluate the EGS derived from different remote sensing data. A simple linear regression was used to analyze the spatial distribution and temporal trends of EGS for each pixel from 2000 to 2018. In this analysis, we also compared the trends of the EGS across different vegetation types.

Previous studies showed that precipitation, minimum temperature and maximum temperature all play an important role in regulating vegetation phenology (Piao et al. 2019; Wang et al. 2019b). A partial correlation analysis was used to evaluate the response of the EGS to climatic factors, including precipitation, *T*_max_, and *T*_min_, during preseason 5 months (with 1 month step). We used the absolute values of the maximum partial correlation coefficients for each pixel to identify the preseason period that was significantly associated with EGS, which was designated as the optimal preseason periods in the study area. Then, we analyzed the relationship between climatic factors and the EGS during the most related preseason periods and determined its significance of each pixel. Using the correlation coefficients, we also assessed the relationship between EGS and climatic factors across different vegetation types.

Finally, to further investigate the response of EGS to climate factors, we conducted multiple regressions to evaluate the sensitivity of EGS to the preseason *T*_max_, *T*_min_ and cumulative precipitation. The coefficients of each factor in the regression model indicate the sensitivity of EGS to corresponding climatic factors. In this study, we aggregated all data to a 0.1° × 0.1° grid to match the coarsest resolution among all datasets. In all the calculation and analysis, we excluded the area with low vegetation coverage (EVI < 0.08) and retained those areas covering the five types of vegetation in the study.

## Results

### Spatial and temporal patterns of EGS in subtropical China

**Fig. 2:**
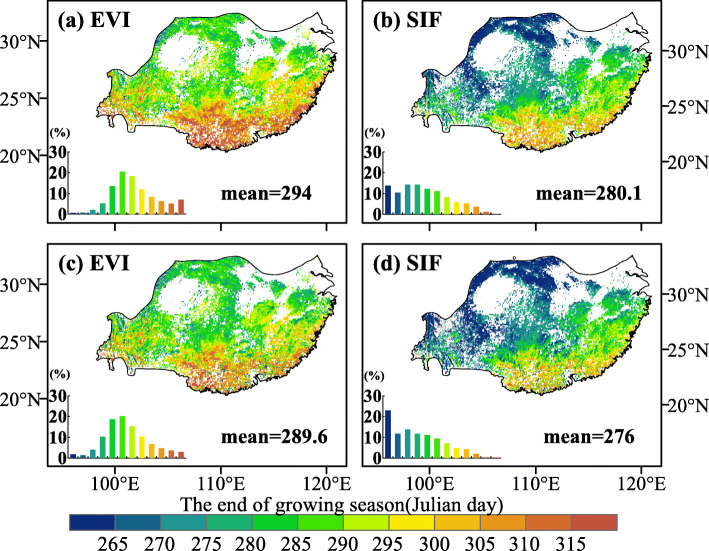
The spatial patterns of the end of growing season (EGS) in subtropical vegetation in China from 2000 to 2018: MODIS EVI dataset and SIF dataset. **a**-**b**, derivative method and **c**-**d**, dynamic threshold method. Inset plots (the bottom-left of each figure) display the frequency distribution of EGS

**Fig. 3:**
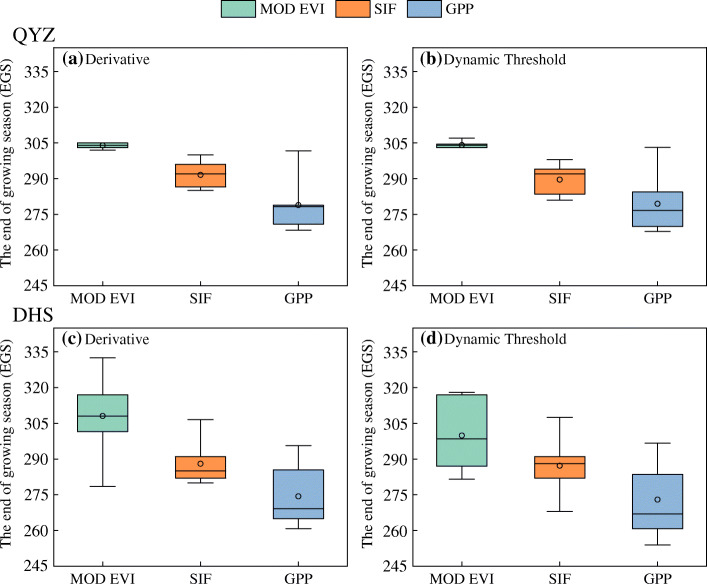
Comparison of the end of growing season (EGS) retrieved from remote sensing data and flux data. Two ecological monitoring stations were listed, including Qianyanzhou station (QYZ) and Dinghushan station (DHS). Both methods were listed, including derivative and dynamic threshold methods

There was a distinct latitudinal variation of EGS derived from SIF and EVI data (Fig. 2): an advance of the EGS at higher latitudes and a delay of the EGS at lower latitudes. Furthermore, EGS extracted from SIF data (EGS_sif_) was earlier than that derived from the EVI data (EGS_evi_). For the two datasets, the spatial distributions of EGS were similar between two methods. In the study area, EGS_sif_ extracted by the derivative and dynamic threshold methods were 280.1 and 276 days, respectively, with an average of 278 days (Fig. 2b and d). In contrast, EGS_evi_ of the two methods were 294 and 289.6 days, respectively, with an average of 291.8 days (Fig. 2a and c). In addition, some interesting information was found by comparing EGS estimated by the two types of remote sensing data and flux data from 2003 to 2010 in Fig. 3. At the Qianyanzhou station, the EGS of EVI, SIF and GPP flux data (average of 8 years) by the derivative method were on day 303.9, 291.6 and 278.9, respectively (Fig. 3a). EGS_evi_, EGS_sif_ and EGS_GPP_ estimated by the dynamic threshold method were on day 304.1, 289.6 and 279.5, respectively (Fig. 3b). The average values of EGS retrieved from two kinds of remote sensing data were later than EGS_GPP_, and the time-lags were 25 days (EGS_evi_) and 11 days (EGS_sif_), respectively. At the Dinghushan station, EGS_evi_, EGS_sif_ and EGS_GPP_ by the derivative method were on day 300.4, 288 and 273.9, respectively (Fig. 3c). For the dynamic threshold method, EGS_evi_, EGS_sif_ and EGS_GPP_ were on day 299.9, 287.1 and 272.7, respectively (Fig. 3d). The average of EGS_GPP_ was 27 days (EGS_evi_) and 14 days (EGS_sif_) ahead of the two remote sensing data. Compared with SIF and EVI, EGS retrieved by the NIRv was later for the two methods (Supplementary Material: Figs. S1 and S2).

**Fig. 4:**
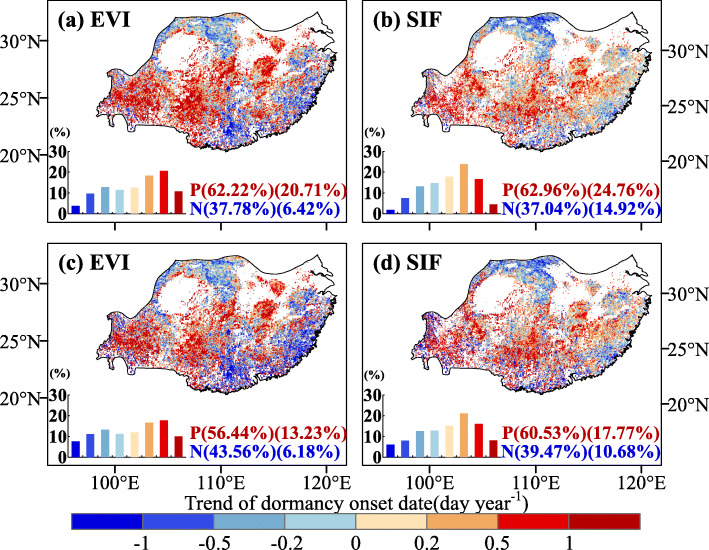
The spatial patterns of the linear trend of the end of growing season (EGS) of subtropical vegetation in China from 2000 to 2018: MODIS EVI dataset and SIF dataset. **a**-**b**, derivative method and **c**-**d**, dynamic threshold method. A negative value indicates an advance, and a positive value indicates a delay. Inset plots (the bottom-left of each figure) display the frequency distribution of change trend. The proportions of positive (P) and negative (N) (proportions of significant in parentheses) trends are provided

Across the study area, the trends in the EGS extracted from the SIF and EVI by the two methods were similar (Fig. 4). In terms of spatial distribution, the delays in EGS_evi_ and EGS_sif_ were restricted to the central part of the study area (Fig. 4). For the derivative method, the delay of EGS_evi_ and EGS_sif_ was observed in more than 60% of the total study area, of which ~ 20% showed significant delays (*P* < 0.05) (Fig. 4a and b). A delay of EGS_evi_ and EGS_sif_ extracted by the dynamic threshold method was observed in more than 55% of the total study area, and the pixels with significant delay (*P* < 0.05) for EGS_evi_ and EGS_sif_ accounted for 13.23%, and 17.77% of all the pixels in the study region, respectively (Fig. 4c, d). Both EGS_evi_ and EGS_sif_ extracted by the two methods exhibited similar trends across different vegetation types. A delayed EGS was observed across most vegetation types (e.g., evergreen forest, shrub, and grassland), except for the deciduous broadleaved forest (Fig. S3). In contrast, the averaged EGS of DBF retrieved from the two methods showed an advancing trend by 0.09 day·yr^− 1^ (EGS_evi_) or 0.37 day·yr^− 1^ (EGS_sif_). In addition, in the study area and different vegetation types, the trend in the EGS extracted from the NIRv was different from that of the SIF and EVI (Figs. S4, S5).

### Response of EGS to climate drivers

Based on the above analysis, the SIF showed a better performance than EVI and NIRv in capturing the EGS, and thus we chose EGS_sif_ to explore the relationship between autumn phenology and climate factors in the study area. At the regional scale, for both two methods, EGS_sif_ in subtropical China was correlated with *T*_min_ during the period of 2–4 months prior to EGS_sif_, the median and mean of the period related to *T*_min_ were in the 3 months prior to EGS_sif_. The mean of the period related to *T*_max_ was in the 2 months to EGS_sif_. For the cumulative precipitation, the EGS_sif_ was most correlated with the period about 3 months prior to EGS_sif_ (Fig. S6).

**Fig. 5:**
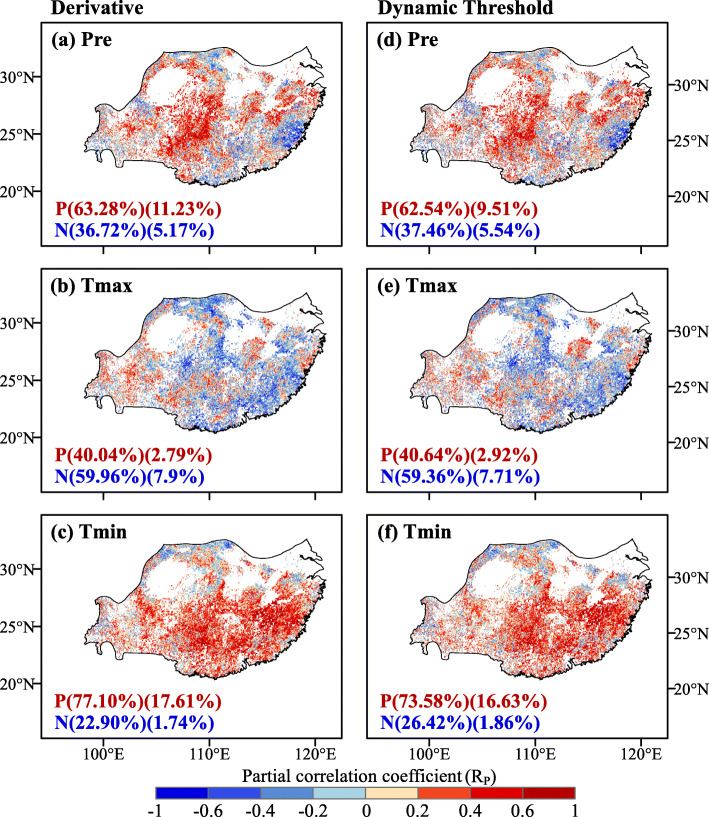
Spatial pattern and frequency distribution of partial correlation coefficient between the end of growing season (EGS_sif_) and climatic factors: **a**, **d** precipitation (Pre), **b**, **e** maximum temperature (*T*_max_) and **c**, **f** minimum temperature (*T*_min_). The methods of the left panel and the right panel are derivative and dynamic threshold methods respectively. The percentages of positive (P) and negative (N) correlations (*P* < 0.05, percentage of significant correlations in parentheses) are provided

Based on the partial correlation analysis, we found that a large proportion of the pixels showed positive correlations between EGS_sif_ extracted by the two methods and *T*_min_ as well as cumulative precipitation (Fig. 5). For the derivative and dynamic threshold methods, about 77.1% and 73.58% of the pixels covering the study area showed a positive partial correlation with *T*_min_, of which 17.61% and 16.63% showed statistically significant relationships (*P* < 0.05), respectively. Similarly, on average, 62.91% of the pixels covering the study area of the two methods showed a positive partial correlation with preseason cumulative precipitation, and this correlation was significant over 9.5% of pixels (*P* < 0.05). Compared with the preseason *T*_min_ and cumulative precipitation, there were more pixels with negative correlation between the preseason *T*_max_ and EGS_sif_, accounting for 59.96% and 59.36% of the study area for the two methods, respectively (Fig. 5).

**Fig. 6:**
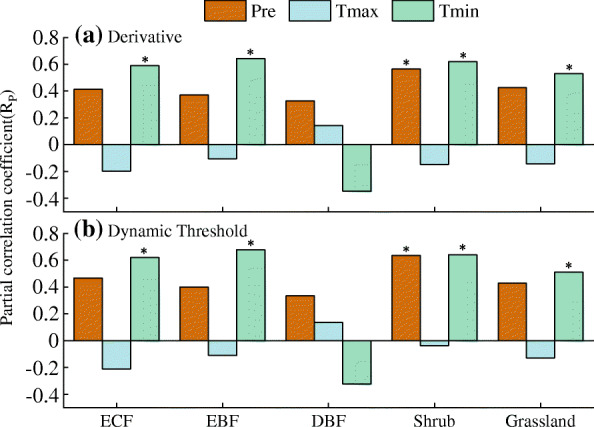
Correlation between the end of growing season (EGS_sif_) of different vegetation and climatic factors. Five main vegetation types in this study area were listed, such as evergreen coniferous forest (ECF), evergreen broadleaved forest (EBF), deciduous broadleaved forest (DBF), shrub, and grassland. **a** derivative method and **b** dynamic threshold method. * indicates statistically significant trends at the 95% (*P* < 0.05) level

The responses of EGS of different vegetation types to climate factors were different (Fig. 6). For two methods, there was a positive partial correlation between EGS_sif_ and *T*_min_ across different vegetation types (*R*_P_ > 0.51, *P* < 0.05; Fig. 6), with the exception of deciduous broadleaved forest (*R*_P_ < − 0.32, *P* > 0.05). Preseason cumulative precipitation was positively correlated with EGS_sif_ for all vegetation types, and the strongest relationship was observed in the shrub (*R*_P_ > 0.56, *P* < 0.05). In contrast, *T*_max_ was negatively correlated with EGS_sif_ across all vegetation types, except for DBF. Overall, EGS_sif_ was more strongly correlated with preseason *T*_min_ and cumulative precipitation than with preseason *T*_max_ (Fig. 6). To further test the results of the partial correlation analysis, we also analyzed the sensitivity of EGS_sif_ to climate factors. Both methods showed similar results. The sensitivity of EGS to the preseason *T*_min_ was the strongest across all vegetation types (> 1.93 day·sd^− 1^), except for DBF (Fig. S7). Conversely, the sensitivity of EGS to the preseason *T*_max_ was the weakest across different vegetation types (Fig. S7).

## Discussion

### Comparison of satellite-retrieved EGS based on SIF and EVI data

By comparing SIF with GPP estimated across two flux tower sites in the study area, the SIF dataset used in this study exhibited strong seasonal and interannual dynamics that were consistent with those of daily GPP (Fig. S8), and thus the reconstructed SIF product has a great potential for monitoring the photosynthetic phonology in the study area. We also found that the SIF clearly had an advanced EGS than did EVI, and it was closer to GPP-derived EGS at the two evergreen forest sites. The differences in the seasonal cycle of EVI and SIF could be explained by the differences in the information contained in the two kinds of data resource. On the one hand, the SIF is deemed to be directly linked to photosynthetic activity, which contains major information on photosynthetically active radiation (Walther et al. 2016). While the EVI is more of an approximation for fraction of photosynthetically active radiation, which indicates the photosynthetic potential of the terrestrial vegetation cover (Jiang et al. 2008). Because of the intrinsic limitations of the photosynthetic machinery and external stress factors, the absorbed photosynthetically active radiation by vegetation cannot be completely used for carbon fixation (Baker 2008). In contrast, SIF contains information on not only absorbed photosynthetically active radiation but also environmental stresses that determine photosynthetic light use efficiency (Yoshida et al. 2015; Li and Xiao 2020). Therefore, SIF can be used to track changes in physiological changes induced by environmental stresses in the absence of changes in greenness or structure. This may explain why EGS based on SIF occurred earlier than that based on the “greenness” phenology reflected in EVI data.

On the other hand, SIF is an energy flux emitted from plant chlorophyll molecules a few nanoseconds after light absorption by vegetation (Baker 2008), whereas the EVI was calculated based on vegetation canopy reflectance. Multiple studies have reported that physiological vegetation indices (e.g., chlorophyll) performance better than structural vegetation indices for detecting the autumn photosynthetic phenology for evergreen forests (Wong et al. 2019; Yin et al. 2020). The structural recession of evergreen forest in autumn is gradual, and the photosynthetic rate is mainly controlled by physiology (Gallinat et al. 2015). Our result indicated that autumn photosynthesis in subtropical China forests is mainly stressed by minimum temperature variability, which causes photosynthesis to end before structural recession (Jeong et al. 2017). Therefore, SIF outperformed EVI in detecting the photosynthetic phenology. In addition, although EVI is an advanced vegetation index, it is still affected by clouds and other atmospheric noise (Miura et al. 2001; Huete et al. 2002). In contrast, the SIF is not sensitive to the influence of cloud and atmosphere (Joiner et al. 2014). This may also contribute to the difference in phenology monitoring for SIF and EVI in our study.

### Response of vegetation phenology to climate drivers

Different from the earlier spring phenology which was mainly caused by global warming, there is no consistent conclusion on the change trend of EGS and its influencing factors. Therefore, it is difficult to explain the response mechanism of EGS to climate change. Especially for the photosynthesis phenology in the subtropical China, the climate response mechanism of autumn photosynthesis is largely unknown due to the limitation of monitoring methods. Our results indicated that the EGS in subtropical China was slightly delayed, and the change trend of EGS was biome dependent. In temperate regions, multiple studies have also confirmed that the EGS has reported a small delay in recent years; however, there were no widespread delaying trends in autumn phenology (Wang et al. 2019a). Our findings indicated that global climate change can extend the growing season in subtropical vegetation, which can in turn enhance the carbon sink capacity of subtropical vegetation.

Previous studies have shown that temperature plays a key role in regulating vegetation autumn phenology (Cleland et al. 2007; Chuine et al. 2010). For example, the increase of *T*_max_ can advance or delay autumn phenology, while the increase of *T*_min_ had the opposite effect (Wu et al. 2018). Our results indicated that the increase of preseason *T*_min_ significantly delayed autumn photosynthetic phenology, extending the growing season in subtropical vegetation in China. The increase of the preseason *T*_min_ may delay the coloring of leaves in autumn and reduce the chilling injury caused by low nighttime temperature (Yang et al. 2017). Additionally, the increase of *T*_min_ can result in warmer autumn weather, fewer frost days and a delay in the first frost (Liu et al. 2016). In our study, the sensitivity of each vegetation type to the preseason *T*_min_ was stronger. This could be due to the fact that subtropical regions are warm and humid, and the nighttime temperature is a limiting factor for vegetation growth, thereby leading to a greater impact on vegetation growth and development than daytime temperature and precipitation. On the other hand, the increase of the preseason *T*_max_ led to an earlier EGS and shortened the growing season in our study. The higher subtropical daytime temperature can decrease photosynthetic enzyme activity (Rossi et al. 2017), which can in turn inhibit photosynthesis and shorten the growing season. In addition, the increase of daytime temperature can also lead to higher evapotranspiration and lower soil water use efficiency, resulting in earlier senescence of vegetation (Estiarte and Penuelas 2015; Wu et al. 2018).

Our results also indicated that preseason cumulative precipitation had a positive impact on the EGS, resulting in a longer growing season. Water is one of the important components of protoplasm, and the amount of water in vegetation affects its metabolic intensity and photosynthetic rate (Quetin and Swann 2017). The sensitivity of vegetation to water varied with stage of growth (Quetin and Swann 2017). In the subtropical region, the increase of cumulative precipitation will strengthen the absorption of nutrients by vegetation and promote the effective photosynthesis (Bertani et al. 2017), delaying the EGS. Additionally, the impact of precipitation on vegetation phenology in the subtropical region has regional and biological specificity (Zhou et al. 2018). Although there is abundant precipitation in the subtropical region of China, the evergreen forests are more suitable in wet areas. Therefore, the increase of preseason precipitation can enhance the physiological activity of vegetation and photosynthesis, thus delaying the vegetation autumn phenology.

It should be noted that the preseason *T*_max_ and *T*_min_ had opposite effects on EGS in deciduous broadleaved forests in the study area. Wu et al. (2018) also reported that the *T*_max_ and *T*_min_ may have the opposite effects on vegetation autumn phenology in different regions. The elevated *T*_min_ can increase nighttime respiration and consumption of organic compounds, leading to a shorter growing season; in contrast, higher *T*_max_ promotes photosynthesis and delays the EGS (Wu et al. 2018). We speculated that the increase of the maximum temperature delayed the EGS, which may be related to the higher latitude position of deciduous broadleaved forest. In this region, the temperature is relatively low, and the increase of the maximum temperature is beneficial for photosynthesis. Our results also indicated that the effect of minimum temperature on EGS was stronger than that of maximum temperature, which may provide an explanation for the advancing trend of EGS in deciduous broadleaved forest. Another possible explanation is that there had been a slight decrease in rainfall across the deciduous broadleaved forest region over the past two decades (Table S1), which may inhibit the extension of the vegetation growing season. Therefore, the comprehensive effect of climate factors on EGS could partly explain the advancing trend in EGS for deciduous broadleaved forest.

### Implications

Using remote sensing data to retrieve the phenological information of deciduous forest has been well reported (Yang et al. 2015; Liu et al. 2016), while the research on phenological monitoring of evergreen forest was still scarce. Our findings indicated that the remote sensing phenological monitoring based on SIF was closer to the photosynthetic phenology in subtropical vegetation. Our results confirmed a lag between the autumn decrease of photosynthesis and the change in greenness in evergreen forests (Walther et al. 2016). This implies that estimates of the EGS purely based on greenness indices will be biased in evergreen forest in subtropical regions, which translates into errors in the autumn carbon budget. Therefore, compared with traditional vegetation indices, SIF can better capture the autumn decrease stage of photosynthesis of subtropical vegetation and effectively improve the dynamic monitoring of photosynthetic activity in evergreen ecosystems of subtropical regions. Our results provide a new reference for the study of subtropical vegetation phenology, and demonstrate the potential of SIF for simulating carbon budget in evergreen ecosystems of subtropical regions.

In the context of global climate change, it needs to be explained, that how vegetation phenology responds to climate change. However, the response mechanisms of autumn phenology of subtropical vegetation to climate change remain unclear. Our results indicated that the effects of precipitation, maximum and minimum temperature on autumn phenology of subtropical vegetation were discrepant, and elucidating this inconsistency is beneficial to the establishment of subtropical vegetation phenology models. Furthermore, our results suggested the most significant effect of the minimum temperature on autumn phenology of subtropical vegetation, which can improve the understanding of the control factors of subtropical vegetation phenology. Based on our study, we proposed to further explore the effects of climate change on autumn phenology of evergreen forests in other regions of the world. This is an important implication for the improvement of phenological parameterization of terrestrial ecosystem models.

## Conclusions

In this study, we used SIF and EVI data to examine the spatial and temporal variation of autumn vegetation phenology and to analyze its response to climatic factors in subtropical vegetation in China. We found that the delay of EGS occurred in evergreen forests, shrub and grassland, but not in the deciduous broadleaved forest. We found that the preseason *T*_min_ and cumulative precipitation were positively associated with the delay of EGS (with positive correlation for more than 73% and 62% of the study areas for the two methods, respectively). Conversely, the preseason *T*_max_ was negatively associated with the EGS (with negative correlation for more than 59% of the study areas for both methods). In all vegetation types except deciduous broadleaved forest, the increase of *T*_min_ also caused the delay of EGS (> 1.93 day·sd^− 1^), while the increase of preseason *T*_max_ advanced it. For precipitation, the increase of cumulative precipitation could delay the EGS across all vegetation types. Our study indicated that the preseason *T*_min_ had a significant effect on the photosynthetic phenology of subtropical evergreen vegetation, providing new insights into how climate change affects the EGS. These results also provide a scientific basis for the development of phenology models for evergreen vegetation.

## Supplementary Information

**Additional file 1: Figure S1.** The spatial patterns of the end of growing season (EGS) in subtropical vegetation in China from 2000 to 2018: NIRv dataset. a) derivative method and b) dynamic threshold method. Inset plots (the bottom-left of each figure) display the frequency distribution of EGS. **Figure S2.** Comparison of the end of growing season (EGS) retrieved from NIRv and flux data. Two ecological monitoring stations were listed, including Qianyanzhou station (QYZ) and Dinghushan station (DHS). Both methods were listed, including derivative and dynamic threshold methods. **Figure S3.** Linear trends of the end of growing season (EGS) across China’s Subtropical biomes from 2000 to 2018. Five main vegetation types in study area were listed, including evergreen coniferous forest (ECF), evergreen broadleaved forest (EBF), deciduous broadleaved forest (DBF), shrub, and grassland. a) derivative and b) dynamic threshold method. A negative value indicates an advance, and a positive value indicates a delay. * indicates statistically significant trends at the 90% (*P* < 0.1) level, ** indicates statistically significant trends at the 95% (*P* < 0.05) level. **Figure S4.** The spatial patterns of the linear trend of the end of growing season (EGS) of subtropical vegetation in China from 2000 to 2018: NIRv dataset. a) derivative method and b) dynamic threshold method. Inset plots (the bottom-left of each figure) display the frequency distribution of change trend. The proportions of positive (P) and negative (N) (proportions of significant in parentheses) trends are provided. **Figure S5.** Linear trends of the end of growing season (EGS) across China’s Subtropical biomes from 2000 to 2018: NIRv dataset. Five main vegetation types in study area were listed, including evergreen coniferous forest (ECF), evergreen broadleaved forest (EBF), deciduous broadleaved forest (DBF), shrub, and grassland. a) derivative and b) dynamic threshold method. A negative value indicates an advance, and a positive value indicates a delay. * indicates statistically significant trends at the 90% (*P* < 0.1) level, ** indicates statistically significant trends at the 95% (*P* < 0.05) level. **Figure S6.** Optimal preseason periods depicting correlations between the end of growing season (EGS) derived from SIF data and climatic factors: Precipitation (Pre), maximum temperature (*T*_max_) and minimum temperature (*T*_min_). Two methods (derivative and dynamic threshold methods) were listed. **Figure S7.** Sensitivity of end of growing season (EGS) to climatic factors in different vegetation types. Five main vegetation types in study area were listed, including evergreen coniferous forest (ECF), evergreen broadleaved forest (EBF), deciduous broadleaved forest (DBF), shrub, and grassland. a) derivative and b) dynamic threshold method.1 day·sd^− 1^ denoted that an increase of 1 standard deviation (sd) in the climatic factors delayed or advanced the EGS by 1 day. **Figure S8.** The seasonal cycles of SIF and flux tower GPP from 2003 to 2010. **Table S1.** The change rate and significance of three climate factors in different vegetation areas from 2000 to 2018, including precipitation (Pre), maximum temperature (T_max_) and minimum temperature (T_min_). Five main biomes in this study area were listed, such as evergreen coniferous forest (ECF), evergreen broadleaved forest (EBF), deciduous broadleaved forest (DBF), shrub, and grassland.

## Data Availability

The GOSIF product is available from https://globalecology.unh.edu/data.html. The MODIS EVI product analysed during the current study is available in the NASA, https://ladsweb.modaps.eosdis.nasa.gov/search/. The meteorological data is available from https://data.tpdc.ac.cn/zh-hans/. The flux data is available in the national flux network of China, http://www.cern.ac.cn/0index/index.asp. Other datasets used during the current study are available from the authors on reasonable request.

## Abbreviations

SIF: Solar-induced Chlorophyll Fluorescence
EVI: Enhanced Vegetation Index
NIRv: Near-infrared Reflectance of Vegetation
EGS: The End of the Growing Season
SOS: The Start of the Growing Season
ECF: Evergreen Coniferous Forest
EBF: Evergreen Broadleaved Forest
DBF: Deciduous Broadleaved Forest
*T*_max_: Maximum Temperature
*T*_min_: Minimum Temperature
GPP: Gross Primary Productivity
NDVI: Normalized Difference Vegetation Index
WPL: Webb-Pearman-Leuning
