# Supplementary material for: Strong controls of daily minimum temperature on the autumn photosynthetic phenology of subtropical vegetation in China
Source: For Ecosyst. 2021 May 19;8(1):31. doi: 10.1186/s40663-021-00309-9 (PMC8550766; doi:10.1186/s40663-021-00309-9)
Supplement: Supplementary file 1 — Additional file 1: Figure S1. The spatial patterns of the end of growing season (EGS) in subtropical vegetation in China from 2000 to 2018: NIRv dataset. a) derivative method and b) dynamic threshold method. Inset plots (the bottom-left of each figure) display the frequency distribution of EGS. Figure S2. Comparison of the end of growing season (EGS) retrieved from NIRv and flux data. Two ecological monitoring stations were listed, including Qianyanzhou station (QYZ) and Dinghushan station (DHS). Both methods were listed, including derivative and dynamic threshold methods. Figure S3. Linear trends of the end of growing season (EGS) across China’s Subtropical biomes from 2000 to 2018. Five main vegetation types in study area were listed, including evergreen coniferous forest (ECF), evergreen broadleaved forest (EBF), deciduous broadleaved forest (DBF), shrub, and grassland. a) derivative and b) dynamic threshold method. A negative value indicates an advance, and a positive value indicates a delay. * indicates statistically significant trends at the 90% (P < 0.1) level, ** indicates statistically significant trends at the 95% (P < 0.05) level. Figure S4. The spatial patterns of the linear trend of the end of growing season (EGS) of subtropical vegetation in China from 2000 to 2018: NIRv dataset. a) derivative method and b) dynamic threshold method. Inset plots (the bottom-left of each figure) display the frequency distribution of change trend. The proportions of positive (P) and negative (N) (proportions of significant in parentheses) trends are provided. Figure S5. Linear trends of the end of growing season (EGS) across China’s Subtropical biomes from 2000 to 2018: NIRv dataset. Five main vegetation types in study area were listed, including evergreen coniferous forest (ECF), evergreen broadleaved forest (EBF), deciduous broadleaved forest (DBF), shrub, and grassland. a) derivative and b) dynamic threshold method. A negative value indicates an advance, and a positive [file 40663_2021_309_MOESM1_ESM.docx]

**Supplementary Material**

**
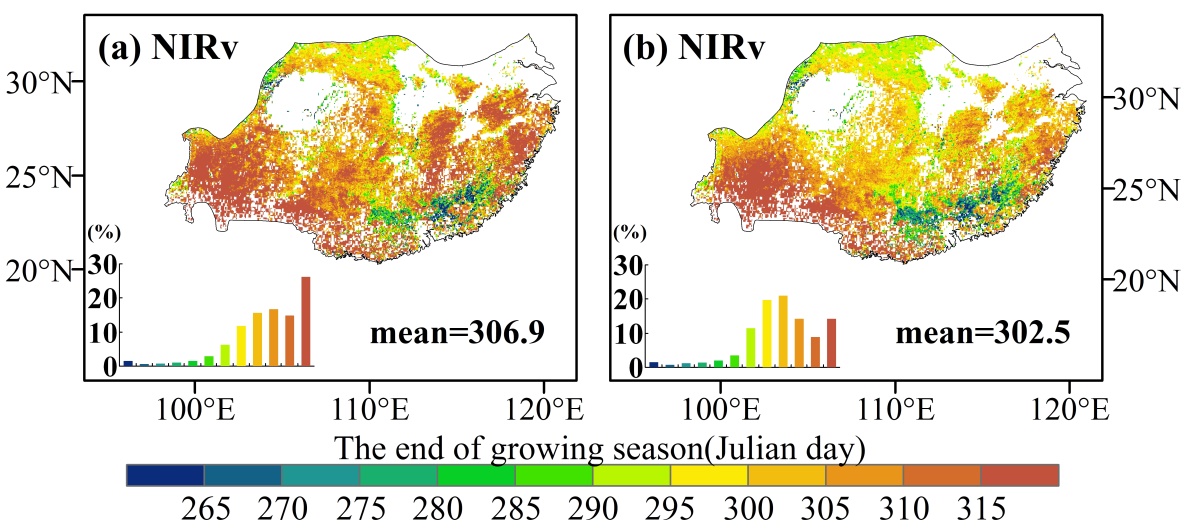
**

**Fig. S1** The spatial patterns of the end of growing season (EGS) in subtropical vegetation in China from 2000 to 2018: NIRv dataset. a) derivative method and b) dynamic threshold method. Inset plots (the bottom-left of each figure) display the frequency distribution of EGS.


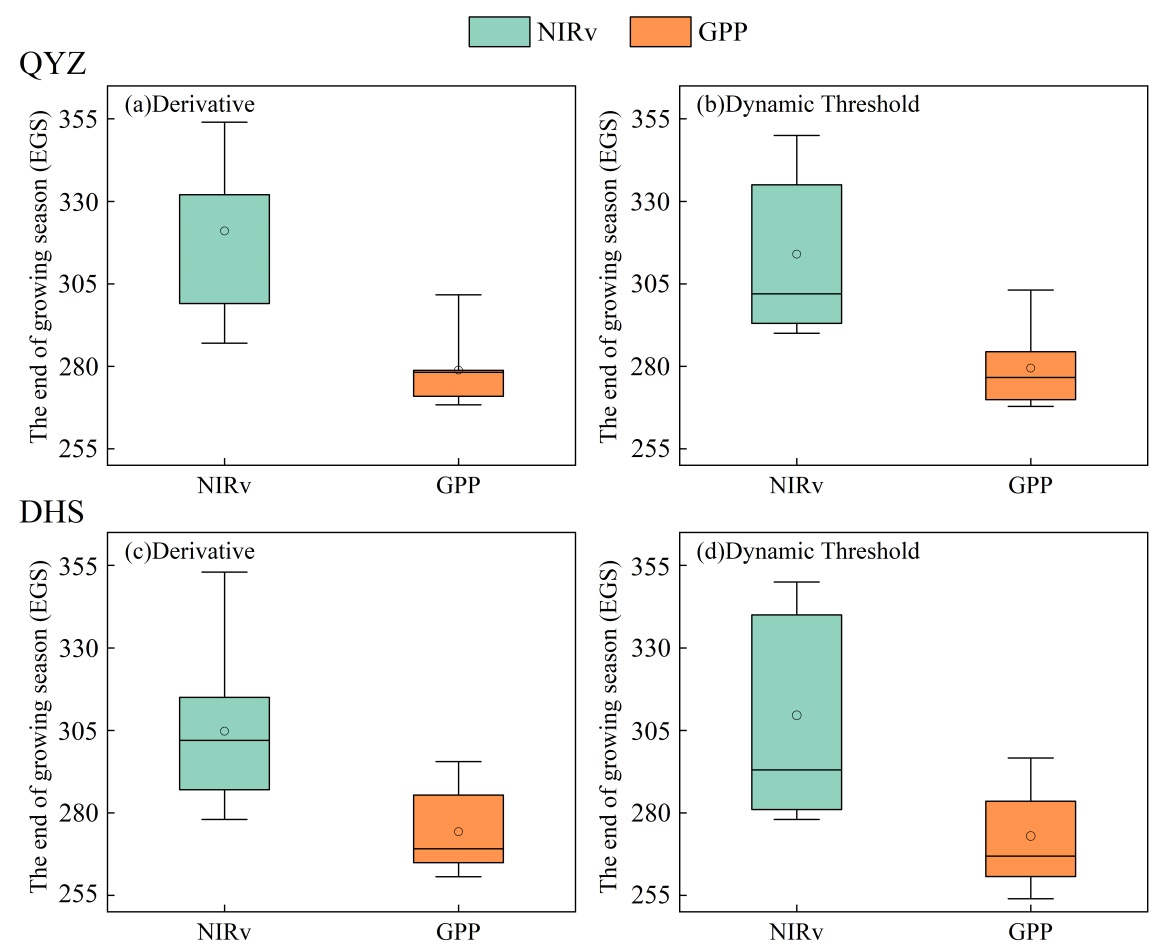


**Fig. S2** Comparison of the end of growing season (EGS) retrieved from NIRv and flux data. Two ecological monitoring stations were listed, including Qianyanzhou station (QYZ) and Dinghushan station (DHS). Both methods were listed, including derivative and dynamic threshold methods.


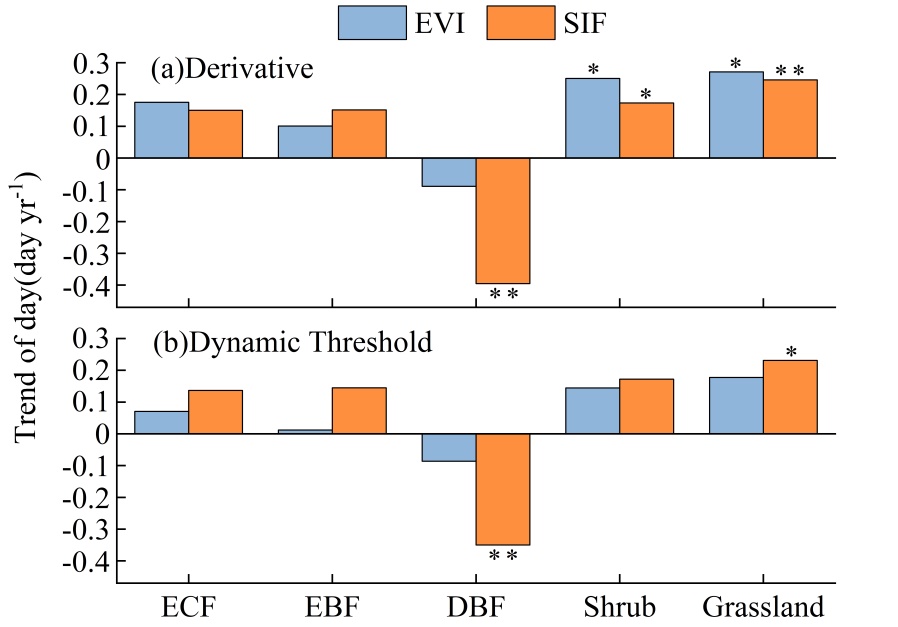


**Fig. S3** Linear trends of the end of growing season (EGS) across China’s Subtropical biomes from 2000 to 2018. Five main vegetation types in study area were listed, including evergreen coniferous forest (ECF), evergreen broadleaved forest (EBF), deciduous broadleaved forest (DBF), shrub, and grassland. a) derivative and b) dynamic threshold methods. A negative value indicates an advance, and a positive value indicates a delay. * indicates statistically signiﬁcant trends at the 90% (*P* < 0.1) level, ** indicates statistically signiﬁcant trends at the 95% (*P* < 0.05) level.


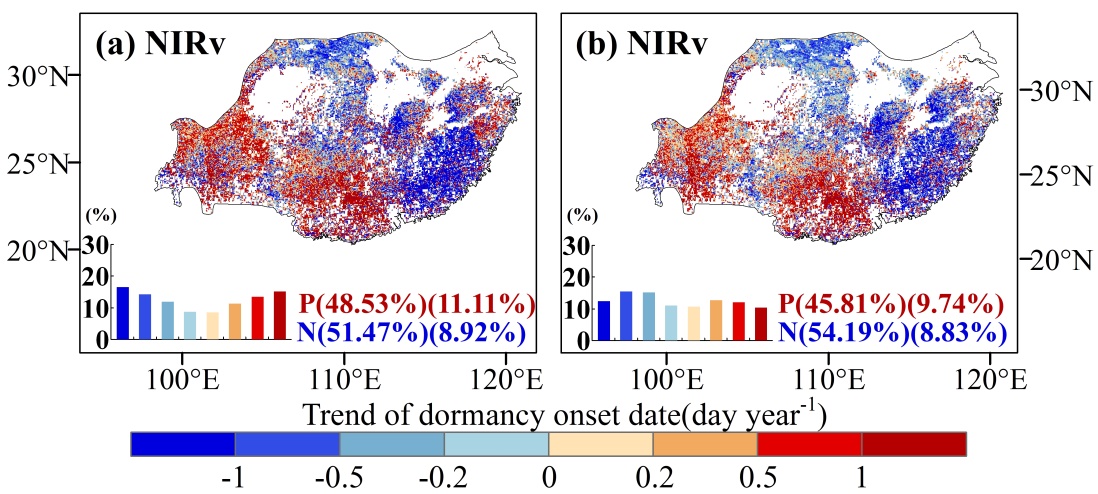


**Fig. S4** The spatial patterns of the linear trend of the end of growing season (EGS) of subtropical vegetation in China from 2000 to 2018: NIRv dataset. a) derivative method and b) dynamic threshold method. Inset plots (the bottom-left of each figure) display the frequency distribution of change trend. The proportions of positive (P) and negative (N) (proportions of significant in parentheses) trends are provided.


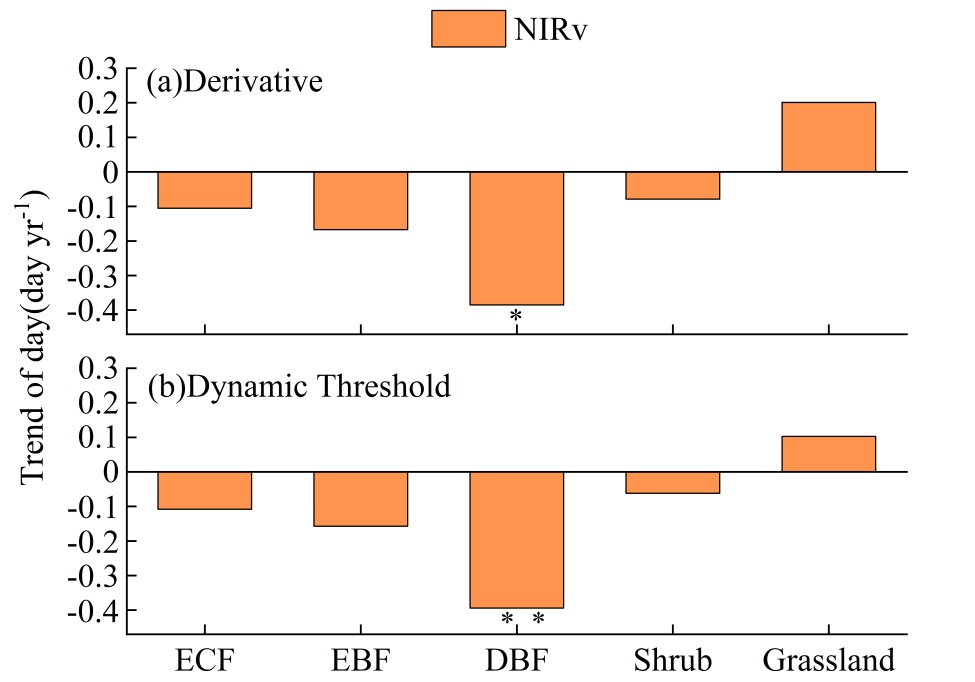


**Fig. S5** Linear trends of the end of growing season (EGS) across China’s Subtropical biomes from 2000 to 2018: NIRv datasets. Five main vegetation types in study area were listed, including evergreen coniferous forest (ECF), evergreen broadleaved forest (EBF), deciduous broadleaved forest (DBF), shrub, and grassland. a) derivative and b) dynamic threshold methods. A negative value indicates an advance, and a positive value indicates a delay. * indicates statistically signiﬁcant trends at the 90% (*P* < 0.1) level, ** indicates statistically signiﬁcant trends at the 95% (*P* < 0.05) level.


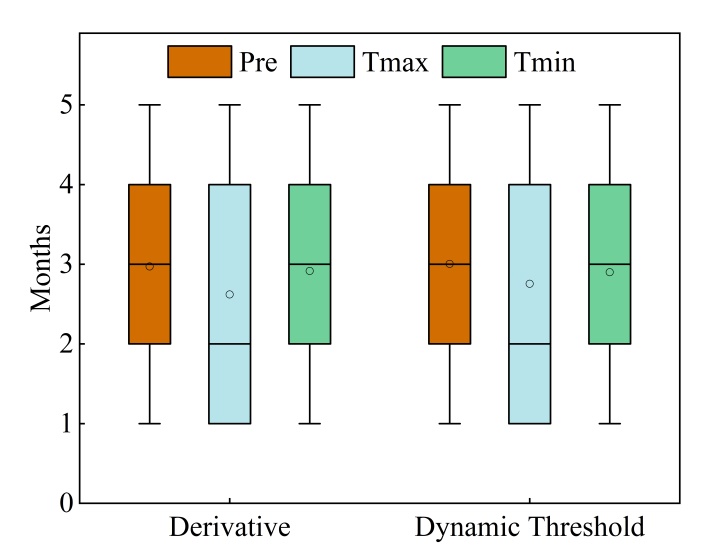


**Fig. S6** Optimal preseason periods depicting correlations between the end of growing season (EGS) derived from SIF data and climatic factors: Precipitation (Pre), maximum temperature (*T*_max_) and minimum temperature (*T*_min_). Two methods (derivative and dynamic threshold methods) were listed.


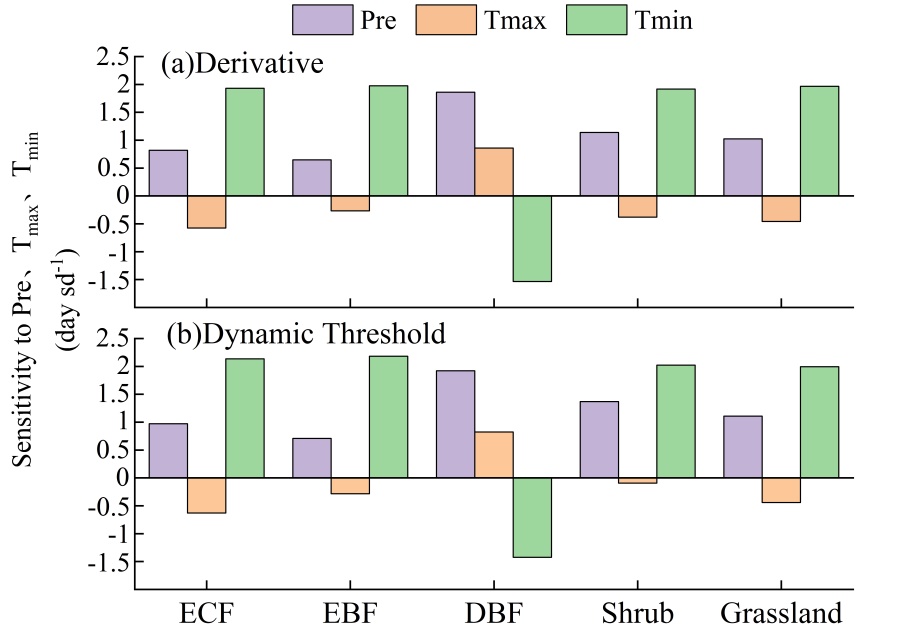


**Fig. S7** Sensitivity of end of growing season (EGS) to climatic factors in different vegetation types. Five main vegetation types in study area were listed, including evergreen coniferous forest (ECF), evergreen broadleaved forest (EBF), deciduous broadleaved forest (DBF), shrub, and grassland. a) derivative and b) dynamic threshold methods.1 day·sd^‒1^ denoted that an increase of 1 standard deviation (sd) in the climatic factors delayed or advanced the EGS by 1 day.


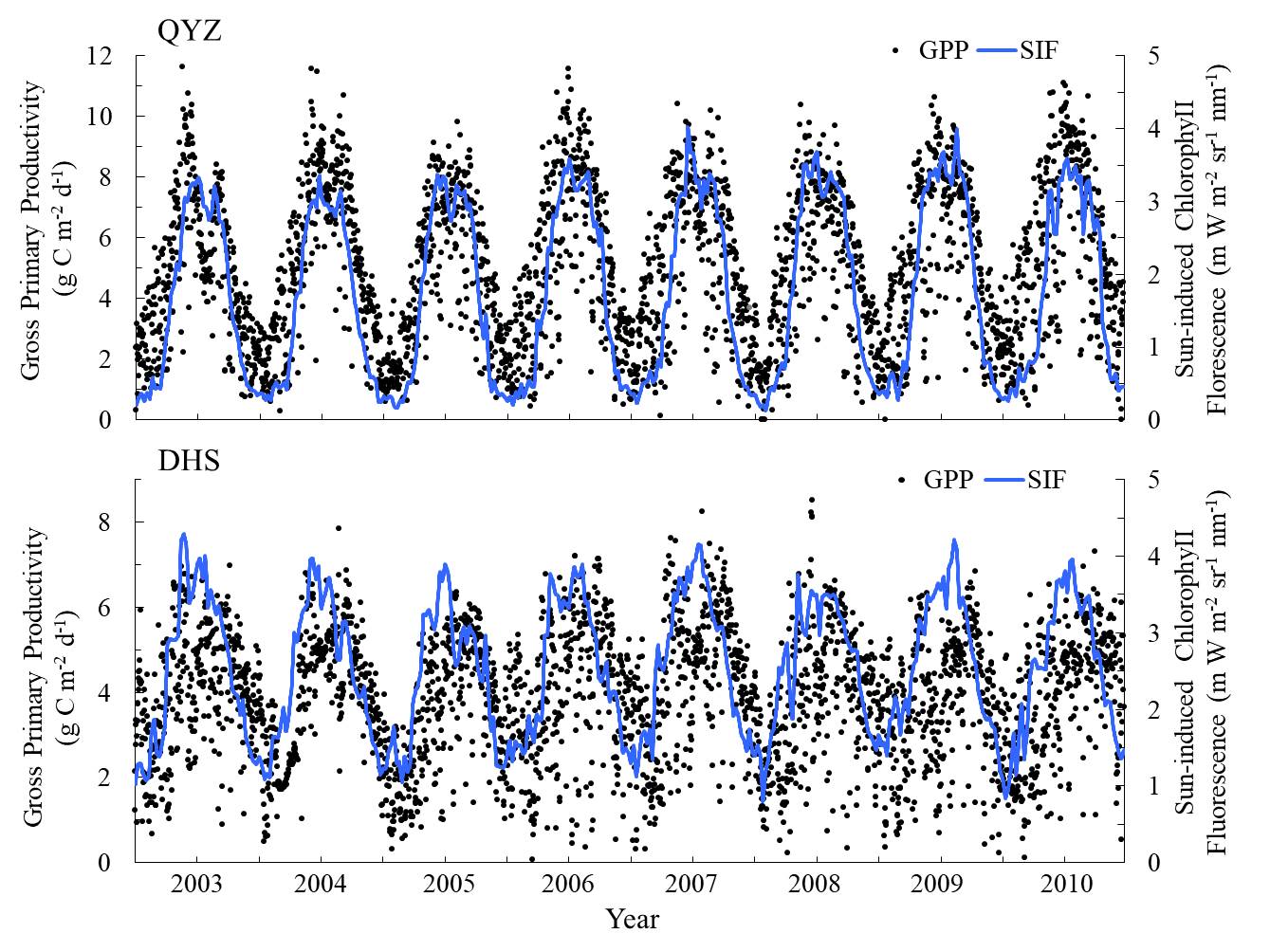


**Fig. S8** The seasonal cycles of SIF and flux tower GPP from 2003 to 2010.

**Table S1** The change rate and significance of three climate factors in different vegetation areas from 2000-2018, including precipitation (Pre), maximum temperature (T_max_) and minimum temperature (T_min_). Five main biomes in this study area were listed, such as evergreen coniferous forest (ECF), evergreen broadleaved forest (EBF), deciduous broadleaved forest (DBF), shrub, and grassland.

| Vegetation types | Rate of change | | |  | Sig. | | |
| --- | --- | --- | --- | --- | --- | --- | --- |
|  | Pre (mm·yr^‒1^) | *T*_max_ (℃·yr^‒1^) | *T*_min_ (℃·yr^‒1^) |  | Pre | *T*_max_ | *T*_min_ |
| ECF | 2.7446 | 0.0408 | 0.0400 |  | 0.4278 | 0.0402 | 0.0479 |
| EBF | 2.4759 | 0.0444 | 0.0447 |  | 0.4890 | 0.0307 | 0.0293 |
| DBF | ‒0.0018 | 0.0679 | 0.0521 |  | 0.9998 | 0.1415 | 0.0321 |
| Shrub | 3.7088 | 0.0353 | 0.0388 |  | 0.3432 | 0.0685 | 0.0496 |
| Grassland | 1.8216 | 0.0491 | 0.0368 |  | 0.5287 | 0.0045 | 0.0498 |
